# Unveiling the challenges of UTUC biopsies and cytology: insights from a global real-world practice study

**DOI:** 10.1007/s00345-024-04866-w

**Published:** 2024-03-20

**Authors:** Joyce Baard, Luigi Cormio, Ranan Dasgupta, Daniele Maruzzi, Soroush Rais-Bahrami, Alvaro Serrano, Bogdan Geavlete, Stilianos Giannakopoulos, Jean de la Rosette, Pilar Laguna

**Affiliations:** 1 Department of Urology, Amsterdam UMC, University of Amsterdam, Amsterdam, The Netherlands; 2 Cancer Center Amsterdam, Amsterdam UMC, Amsterdam, The Netherlands; 3 Department of Urology, University of Foggia, Foggia, Italy; 4 Department of Urology, Imperial College Healthcare NHS Trust, London, UK; 5 Department of Urology, S. Maria Degli Angeli Hospital, Pordenone, Italy; 6 Departments of Urology and Radiology, O’Neal Comprehensive Cancer Center at UAB, University of Alabama at Birmingham, Birmingham, AL USA; 7 Department of Urology, Hospital Clínico San Carlos, Madrid, Spain; 8 Department of Urology, Sanador Hospital, Bucharest, Romania; 9 Department of Urology, School of Medicine, Democritus University of Thrace, University Hospital of Alexandroupolis, Alexandroupolis, Greece; 10 Department of Urology, Istanbul Medipol Mega University Hospital, Istanbul Medipol University, Istanbul, Turkey

**Keywords:** Biopsy, Cytology, KSS, Upper tract urothelial cancer, Ureteroscopy

## Abstract

**Purpose:**

Diagnostic ureteroscopy (dURS) is optional in the assessment of patients with upper tract urothelial carcinoma (UTUC) and provides the possibility of obtaining histology.

**Methods:**

To evaluate endoscopic biopsy techniques and outcomes, we assessed data from patients from the CROES-UTUC registry. The registry includes multicenter prospective collected data on diagnosis and management of patients suspected having UTUC.

**Results:**

We assessed 2380 patients from 101 centers. dURS with biopsy was performed in 31.6% of patients. The quality of samples was sufficient for diagnosis in 83.5% of cases. There was no significant association between biopsy techniques and quality (*p* = 0.458). High-grade biopsy accurately predicted high-grade disease in 95.7% and high-risk stage disease in 86%. In ureteroscopic low-grade tumours, the prediction of subsequent low-grade disease was 66.9% and low-risk stage Ta-disease 35.8%. Ureteroscopic staging correctly predicted non-invasive Ta-disease and ≥ T1 disease in 48.9% and 47.9% of patients, respectively. Cytology outcomes did not provide additional value in predicting tumour grade.

**Conclusion:**

Biopsy results adequately predict high-grade and high-risk disease, but approximately one-third of patients are under-staged. Two-thirds of patients with low-grade URS-biopsy have high-risk stage disease, highlighting the need for improved diagnostics to better assess patient risk and guide treatment decisions.

Clinical trial registration: The study was registered at ClinicalTrials.gov (ClinicalTrials.gov NCT02281188; https://clinicaltrials.gov/ct2/show/NCT02281188).

**Supplementary Information:**

The online version contains supplementary material available at 10.1007/s00345-024-04866-w.

## Introduction

The fundamental basis of treatment strategies for oncological diseases lies in the histologic confirmation and staging of the disease. This is no different for upper tract urothelial carcinoma (UTUC). Both the European Association of Urology (EAU) and the recently published guideline of the American Urology Association (AUA) emphasize the significance of stratifying patients into low- or high-risk disease, based on patient and tumour characteristics. It is important to note that both guidelines utilize a different non-validated risk stratification method, but ultimately with the same conclusion [1, 2]. Patients with low-risk disease may be offered kidney-sparing surgery (KSS) by ureteroscopy (URS) and patients with high-risk disease are indicated for radical surgery by radical nephroureterectomy (RNU).

Both guidelines include a diagnostic URS (dURS) in the diagnostic algorithm. According to the latest revised EAU guideline a dURS should be performed when the diagnosis and risk stratification cannot be reached based on CT imaging or voided urine cytology and preferably without a biopsy due to the high reported intravesical recurrences (IVR) after dURS combined with biopsies [1]. The AUA guideline states that patients suspected of having a UTUC after imaging should be evaluated by endoscopy (antegrade or retrograde approach) *with* tissue sampling and cytologic washing when diagnostic and prognostic details are needed. They emphasize that the diagnostic procedure should be performed as a standardized endoscopic examination including elements pertinent to clinical decision-making and provide a handhold for standardized documentation for reporting of these elements.

Real-world data from the CROES-UTUC registry has shown that a dURS combined with tumour biopsy is not routinely performed by urologists [3]. The diagnostic predictive value of dURS biopsy and the factors that influence its diagnostic yield and accuracy are unclear. Additionally, the prediction of urine cytology in UTUC is known to be poor [3].

The aims of this study were to assess the techniques and outcomes of URS biopsy and their outcomes in patients with suspicion of UTUC and to assess the diagnostic yield of cytology and its added value to dURS and biopsy in UTUC patients in real practice.

## Patients and methods

Data from the CROES-UTUC registry was queried for patients with UTUC that received a biopsy during dURS. The registry includes multicenter prospective collected data on diagnosis and management of adult patients with suspicion of UTUC. The study was registered at ClinicalTrials.gov (ClinicalTrials.gov NCT02281188); a detailed description of this registry protocol has been previously published [4].

In the subpopulation of patients receiving subsequent surgery by RNU or segmental ureter resection (SUR), we compared the biopsy results with the histopathology of the surgical specimen (standard for comparison) (Supplement Fig. 1).

### Study objectives

Our primary focus involved the evaluation of the endoscopic biopsy techniques and their diagnostic yield. Additionally, we evaluated the quality of biopsy specimen stratified by biopsy technique (forceps vs. basket) and evaluated the accuracy of endoscopic biopsy in determining tumour stage and grade. Finally, we assessed the accuracy of cytology results in comparison to histopathology findings, particularly with regard to tumour grade.

### Statistical analysis

All data were analyzed using SPSS version 27 (IBM Corporation Armonk, New York) and summarized by descriptive statistics. Differences between patient groups and quality of biopsy specimen stratified by biopsy technique were assessed by Chi-square tests. Association between the clinical grade and stage prediction and final surgical pathologic outcomes was tested by Fisher’s exact test. The accuracy of biopsy outcomes and cytology (either voided or selective urine samples) or combination of both were evaluated by crosstabs. When combining cytology and biopsy outcomes, we defined low-grade disease when cytology and biopsy outcome was low-grade and high-grade disease when *either* cytology or biopsy outcome was high grade based on clinical relevance.

## Results

During the study period (November 2014–November 2019), 2380 patients from 101 centers (37 countries) were identified. Characteristic of the patients included in the registry have been described elsewhere [3, 5].

Overall, 1184 patients (49.7%) received a dURS, combined with biopsy during the same procedure in 752 patients (31.6% of all patients and 63% of those receiving dURS). The dURS with biopsy sub-cohort was selected for further analysis to answer our primary objective (Supplement Fig. 1).

There were substantial differences in the proportion of tumour focality, size, sign of invasive disease and the use of urine cytology between patients with and without dURS evaluation (Table 1).

**Table 1:** Basic characteristics of patients evaluated with and without ureteroscopic evaluation

	Ureteroscopic evaluation (%)		Chi-square *p*-value
Total *n* = 2380 Missing 230 (9.7%)	Yes 1184 (49.7)	No 966 (40.6)	
Age groups			0.056
< 70 year	566 (47.8)	421 (43.6)	
> 70 year	617 (52.1)	542 (56.1)	
Missing	1 (0.1)	3 (0.3)	
Gender			0.061
Female	369 (31.2)	265 (27.4)	
Male	814 (68.8)	699 (72.4)	
Missing	1 (0.1)	2 (0.2)	
CKD level			0.071
Normal (grade 1 and 2)	637 (53.8)	489 (50.6)	
Low (grade 3–5)	407 (34.4)	370 (38.3)	
Missing	140 (11.8)	107 (11.1)	
Tumour focality			< 0.001
Unifocal	788 (66.6)	693 (71.7)	
Multifocal	117 (9.9)	175 (18.1)	
Missing	279 (23.6)	98 (10.1)	
Tumour size			< 0.001
< 2 cm	384 (32.4)	220 (22.8)	
> 2cm	537 (45.4)	463 (47.9)	
Missing	263 (22.2)	283 (29.3)	
Signs of invasive disease on CT imaging			0.000
Yes	385 (32.5)	533 (55.2)	
No	762 (64.4)	390 (40.4)	
Missing	37 (3.1)	43 (4.5)	
Cytology performed			< 0.001
Yes	657 (55.5)	457 (47.3)	
No	424 (35.8)	418 (43.3)	
Missing	103 (8.7)	91 (9.4)	

Significant differences in proportions were observed between the population who received a dURS-*with biopsy* compared to the group with dURS*-only* for the variables: tumour size and signs of invasive disease (both *p* < 0.001). The population that received endoscopic biopsy consisted of a larger proportion of patients who received a dURS not only as a diagnostic tool, but also as treatment during the same procedure (*p* < 0.001) (Table 2).

**Table 2:** Characteristics of ureteroscopic evaluated patients with and without endoscopic biopsy and technique of URS biopsy

	dURS *only*	dURS *with biopsy*	Chi-square *p*-value
Total patients evaluated with URS *n* = 1184 Missing data *n* = 84 (7.1%)	348 (29.4)	752 (63.5)	
Age groups			0.655
< 70 year	170 (48.9)	356 (47.3)	
> 70 year	178 (51.1)	395 (52.5)	
Missing	0	1 (0.1)	
Gender			0.452
Female	102 (29.3)	238 (31.6)	
Male	245 (70.4)	514 (68.4)	
Missing	1 (0.3)	0	
CKD level			0.227
Normal (grade 1 and 2)	200 (57.5)	411 (54.7)	
Low (grade 3–5)	115 (33.0)	280 (37.2)	
Missing	33 (9.5)	61 (8.1)	
URS goal			< 0.001
Diagnostic only	294 (84.5)	466 (72.0)	
Diagnostic and treatment	51 (14.7)	283 (37.6)	
Missing	3 (0.9)	3 (0.4)	
Tumour focality			0.881
Unifocal	247 (71.0)	510 (67.8)	
Multifocal	38 (10.9)	76 (10.1)	
Missing	63 (18.1)	166 (22.1)	
Tumour size			< 0.001
< 2 cm	154 (44.3)	423 (56.3)	
> 2cm	95 (27.3)	139 (18.4)	
Missing	99 (28.4)	190 (25.3)	
Signs of invasive disease on CT imaging			< 0.001
Yes	162 (46.6)	209 (27.8)	
No	174 (50.0)	521 (69.3)	
Missing	12 (3.4)	22 (2.9)	
Technique of dURS with biopsy (n = 752)			
Tumour location	–		–
Ureter		359 (47.7)	
Pyelocaliceal		308 (41.0)	
Missing		85 (11.3)	
Average number of biopsies	–	2	
Biopsy device	–		–
Basket		199 (26.5)	
Flexible forceps		217 (28.9)	
Rigid forceps		194 (25.8)	
Missing		142 (18.9)	
Type of scope	–		–
Flexible		159 (21.1)	
Semi-rigid		328 (43.6)	
Both		224 (29.8)	
Missing		41 (5.5)	

### Technique of endoscopic biopsies and outcomes

In 83.5% (*n* = 628) of biopsy samples, the quality of the biopsy was sufficient to make a diagnosis. In 55 (7.3%) biopsy samples, there was no specimen found (*n* = 7, 1%) or inadequate material (*n* = 48, 6.4%) for diagnostic purposes. We found no association between the type of ureteroscope used (semirigid/flexible ureteroscope or combination), different biopsy techniques (basket versus flexible and rigid forceps) or the number of biopsies taken and the quality of the biopsy specimen (respectively, *p* = 0.543, *p* = 0.458 and *p* = 0.471). Ureter tumours were more often biopsied by rigid forceps and pyelocaliceal tumours by baskets. Large tumours (> 2 cm) were more often biopsied by baskets compared to flexible and rigid forceps (*p* < 0.005). Of the 628 biopsy samples reported to be of sufficient quality, 404 (64.3%) cases were reported as low-grade tumour, 147 (23.4%) as high grade and in the remaining 77 (12.3%) no information on tumour grade was provided.

Tumour stage was reported in 466/752 biopsies (62%). Most tumour samples were diagnosed as Ta-disease (*n* = 325), followed by T1 (*n* = 81), T2 (*n* = 32), T3 (*n* = 10), T4 (*n* = 1) and Tis (*n* = 17). In 452 cases (452/628, 72%), the pathologist reported on both grade and stage.

### Correlation of clinical and pathological tumour grade

A sub-cohort of 332 patients (332/752, 44.1%) evaluated by dURS and biopsy were treated by either RNU or SUR (Supplement Fig. 1).

### Cytology

In 116 cases, there was information on urine cytology outcome and final surgical grade (Supplement Fig. 1). The urine cytology outcome was concordant with the final pathology grade (*p* = 0.031) and correctly predicted low-grade tumours in 61.5% (8/13) of cases and high-grade tumours in 72.1% (31/43) (Supplement Fig. 2). However, the largest group of patients with information on urine cytology and surgical histology grade were reported as atypical (60/116, 51.7%). In this group, 61.7% (37/60) had high-grade disease in the final specimen.

### Biopsy

In 239 cases, there was information on both endoscopic biopsy grade and final surgical pathology grade (239/332, 72%) (Supplement Fig. 1). The endoscopic biopsy grade was significantly associated with final pathology grade (*p* < 0.001). Endoscopic biopsy outcomes where concordant with low- and high-grade disease in the final specimen in 66.9% (97/145) and in 95.7% (90/94) of cases, respectively. When compared with the surgical specimen, overgrading was seen in 4/94 cases (4.3%) and undergrading in 33.1% (48/145) (Supplement Fig. 2).

When combining the outcomes of urine cytology with the URS biopsy grade, the undergrading was slightly lower (31.5 vs. 33.1%); however, the accuracy of predicting high-grade disease decreased as well to 88.1% (104/118) vs. 95.7% (Supplement Fig. 2).

### Correlation of clinical grade and pathological tumour stage

In 246 cases, there was information on both endoscopic biopsy grade and final surgical pathology stage (74%) (Supplement Fig. 1). Endoscopic low-grade tumours predicted *Ta-disease* in 35.8% cases (54/151). In 4% (6/151), a low-grade biopsied tumour was found to be Tis, and 60% (91/151) of low-grade tumour biopsies had a final tumour stage ≥ T1. A high-grade tumour biopsy predicted high-risk disease (Tis or ≥ T) in 86% (86/95) of cases (Supplement Table 2).

### Correlation biopsy stage and surgical pathologic stage

Information on ureteroscopy tumour stage and final surgical pathology was available in 189/332 cases (56.9%) of the sub-cohort. Ureteroscopic staging of non-invasive Ta-disease and ≥ T1 disease was correctly predicted in 48.9% (46/94) and 47.9% (45/94) of the patients.

High-risk disease (Tis or ≥ T1) on tumour biopsies was correctly predicted in 51.6% (49/95) of cases, but understaged in 47.4% (45/95). In one case, there was overstaging, as a ureteroscopic Tis was found to be a Ta tumour in the final surgical specimen (Supplement Table 2).

## Discussion

Our prospective real-world data on dURS demonstrates that half of the patients with primary suspicion of UTUC received a dURS during initial assessment combined with an endoscopic biopsy in 63.5% of dURS procedures. Overall, the majority of the UTUC biopsies (83.5%) were reported to be of good quality and sufficient for diagnosis, with an excellent prediction of high-grade disease. However, in the population receiving subsequent RNU/SUR, undergrading occurred in one-third of the cases of low-grade diagnostic at biopsy. Conversely high-grade URS biopsy was associated with the presence of invasive (≥ T1) tumour stage.

The subgroup of patients who did not undergo dURS prior to definitive treatment included cases with larger, frequently invasive, and multifocal tumours. In this particular group, the clinical risk assessment was probably mainly influenced by the CT scan results.

Endoscopic biopsies were performed in 63.5% of patients evaluated by dURS. In the subset of patients who did not undergo biopsy, tumours were often larger (> 2 cm) and exhibited a higher incidence of invasive characteristics on CT scans. These observations may suggest that dURS was primarily utilized for diagnostic purposes in this particular group.

In our cohort, there were no differences in diagnostic yield among the three different biopsy devices. We observed that baskets were more often used in case of large (> 2 cm) tumours, likely due to the limited usefulness of baskets in smaller flat lesions. Previous comparison of the diagnostic yield of biopsies taken by stainless steel flat wire baskets versus biopsy forceps shows a significant superiority of the baskets in tissue diagnosis and provision of specific tumour grade (63% vs. 94%, respectively) [6]. A comparison of three different biopsy devices (biopsy forceps 3F and 6F and nitinol basket) resulted in 78.2% of samples being sufficient to set a histological characterization [7]. In that series, all biopsy samples inadequate for diagnosis were taken by biopsy forceps (83.3% and 16.7% using 3F and 6F forceps, respectively). Whilst all the basket biopsies provided a histological diagnosis, the overall number of basket biopsies was very low suggesting a preselection of the instrument used [7].

As described by others, a point of concern in the UTUC endoscopic biopsy is the reliability [8]. Concordance was high between high-grade endoscopic biopsy and high-grade disease in the final surgical specimen (95.7%). However, the correlation of endoscopic and surgical low-grade disease is less favourable. Our 66.9% concordance was similar to those of other reports and entailed that 1/3 of diagnosed low-grade tumours by endoscopy showed upgrading at RNU/SUR and were ultimately high-grade disease [8, 9]. Irrespective of whether this poor concordance arises from issues such as sampling errors, technical challenges, or histopathological variations (such as tumour heterogeneity), these numbers are consistently reported in the literature. It underscores the importance of adhering to strict follow-up protocols and emphasizes the necessity for advancing diagnostic methods to improve patient selection [10, 11].

The accuracy of diagnosing clinical low-grade tumours improved marginally when combined with cytology. Urine cytology was scarcely performed in our cohort and its low additional value reflects the poor sensitivity described by others [3, 12, 13]. Urine cytology correctly diagnosed low-grade disease in only 61.5% of cases and high-grade disease in 72.1%. According to these findings, cytology has no added value when including endoscopic biopsies in the diagnostics algorithm of UTUC.

A last point of attention is the concordance between endoscopic biopsy grade and final tumour stage. Among patients with a low-grade endoscopic biopsy result, only one-third were found to have Ta-stage disease, while the majority had invasive disease or CIS. In the series of Wang et al. [14]; the percentage of patients with definitive invasive UTUC for endoscopic grade 1, 2 and 3 tumours was 38%, 54% and 85%, respectively. Endoscopic biopsy samples are small sized and often do not contain lamina propria; therefore, it seems futile to use the biopsy as a staging tool. Our data further supports the limited value of biopsy in staging, as 60% of the endoscopic biopsy of low-grade disease patients harboured high-risk stage (≥ T1 disease) at the final surgical pathology.

### Clinical implications

Clinical risk stratification aims to differentiate between patients with low- and high-risk disease, to prevent either under- or overtreatment and to determine the need for neoadjuvant systemic therapy. As of now, UTUC guidelines have suggested performing a dURS when imaging and voided cytology do not provide adequate information for diagnosis or risk stratification. According to the revised guideline, this evaluation should ideally be conducted without the use of biopsies. The caution of previous EAU guideline recommendations may be justified by the reported higher intravesical (IVR) therapy after dURS with biopsy [1]. The recent published AUA guideline on diagnosis and management of non-metastatic UTUC strongly recommends dURS *and* biopsy of suspect lesions and cytologic washing of the affected system [2]. While the introduction of new recommendations may alter the diagnostic algorithm for UTUC by incorporating dURS with biopsy, caution is needed to rely on the biopsy for disease *staging* as proven to have limited value in accurately determining the stage of the disease. Our results emphasizes the importance of not only careful selection,but also implementing a rigorous follow-up regimen after endoscopic KSS as 1/3 of diagnosed low-grade tumours shows upgrading.

## Limitations

Although multi-institutional, international and prospectively collected, our data is not devoid of limitations. The lack of a standard diagnostic and treatment protocol resulted in a relatively heterogeneous cohort and the presence of missing data or transcription error (e.g. the unlikely biopsy diagnostic of T2–3) and induce a certain bias that weakens the strength of some conclusions. Any conclusion on the cytology/grade correlation should be regarded with caution because of the small sample. Furthermore, no details were available on sampling techniques. Lastly, a central review was not planned at registry inception and represents a further limitation. Although the literature on UTUC central review is scarce, it is likely that the benefit of central review seen in bladder cancer may be extrapolated to UTUC. Nonetheless, our report allows to determine patterns of practice and realistic and generalizable expectations in terms of endoscopic risk categorization rather than retrospective or isolated reports from reference centres.

## Conclusion

Our real-world data highlights the limitations of the UTUC algorithm in accurately distinguishing patients suitable for KSS or radical surgery. Patients who underwent a full diagnostic evaluation, including dURS and biopsy, were more likely to have clinical suspicion of low-risk disease. Endoscopic biopsy showed a high diagnostic yield for predicting high-grade disease. However, understaging occurred in one-third of patients in the low-grade group, and 60% of patients in the final surgical specimen had high-risk tumours (Tis or ≥ T1 disease). Urine cytology did not provide additional value compared to endoscopic biopsies.

## Supplementary Information

Below is the link to the electronic supplementary material.

Supplementary file 1 (DOCX 100 KB)

## Data Availability

Raw data are under data use agreement and not publicily available.
