# Supplementary material for: Unveiling the challenges of UTUC biopsies and cytology: insights from a global real-world practice study
Source: World J Urol. 2024 Mar 20;42(1):177. doi: 10.1007/s00345-024-04866-w (PMC10954852; doi:10.1007/s00345-024-04866-w)

**Supplement**

Supplement Table 1

| \|  \| **Biopsy device** \| \| \| \| \| --- \| --- \| --- \| --- \| --- \| \| **Basket** \| **Flex forceps** \| **Rigid forceps** \| **Chi square**  **p value** \| \| Location  Ureter  Pyelocaliceal \| 85  100 \| 85  99 \| 126  55 \| <0.005  <0.005 \| \| Tumour size  < 2cm  >2cm \| 54  109 \| 84  85 \| 86  84 \| <0.005  <0.005 \| \| Quality of biopsy  No material or not enough  Enough for diagnosis  Missing data (9.2%) \| 12  173 \| 20  180 \| 16  168 \|  \| |
| --- | --- | --- | --- | --- | --- | --- | --- | --- | --- | --- | --- | --- | --- | --- | --- | --- | --- | --- | --- | --- | --- | --- | --- | --- |

Supplement Table 2 Concordance endoscopic biopsy outcomes and final pathology stage

| **URS biopsy grade** | **Final pathology stage** | | | | | |
| --- | --- | --- | --- | --- | --- | --- |
|  | **Ta** | **Tis** | **T1** | **T2** | **T3** | **T4** |
| **Low grade** | 54/151 (35.8) | 6/151 (4.0) | 34/151 (22.5) | 31/151 (20.5) | 26/151 (17.2) | 0 |
| **High grade** | 9/95 (9.5) | 2/95 (2.1) | 16/95 (16.8) | 17/95 (17.9) | 47/95 (49.5) | 4/95 (4.2) |
|  | | | | | | |
| **URS biopsy stage** | **Final pathology stage** | | | | | |
|  | **Ta** | **Tis** | **T1** | **T2** | **T3** | **T4** |
| **Ta** | **46/94 (48.9)** | 3/94 (3.2) | 20/94 (21.3) | 7/94 (7.4) | 18/94 (19.1) | 0 |
| **Tis** | 1/9 (11.1) | **6/9 (66.7)** | 0 | 0 | 2/9 (22.2) | 0 |
| **T1** | 0 | 0 | **17/56 (30.4)** | 12/56 (21.4) | 24/56 (42.9) | 3/56 (5.4) |
| **T2** | 0 | 0 | 0 | **17/21 (81.0)** | 4/21 (19.0) | 0 |
| **T3** | 0 | 0 | 0 | 0 | **8/8 (100)** | 0 |
| **T4** | 0 | 0 | 0 | 0 | 0 | **1/1(100)** |

**Figures**

Supplement Figure 1

* Due to missing data the analysis were performed on smaller numbers as indicated in the flowchart

Supplement Figure 2 Percentage of over- and under estimation of the different diagnostic outcomes


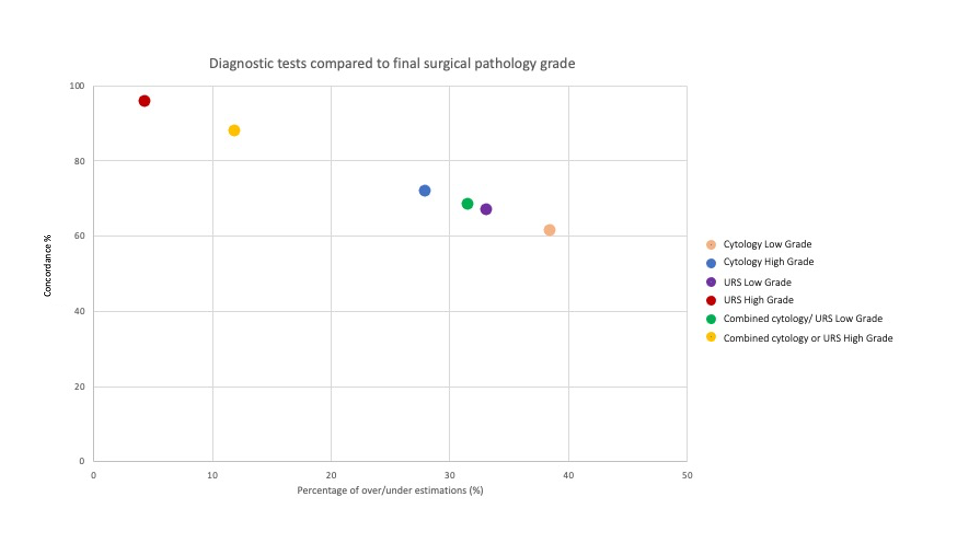

Supplement: Supplementary file 1 — (DOCX 100 KB) [file 345_2024_4866_MOESM1_ESM.docx]
